# Characterization of sperm motility and testosterone secretion in the *taiep* myelin mutant, a model of demyelination

**DOI:** 10.1590/1984-3143-AR2022-0102

**Published:** 2023-10-30

**Authors:** Luz Patricia Muñoz de la Torre, Angélica Trujillo Hernández, José Ramón Eguibar, Carmen Cortés, Leticia Morales-Ledesma

**Affiliations:** 1 Posgrado en Ciencias Biológicas, Universidad Nacional Autónoma de México, Ciudad de México, México; 2 Laboratorio de Fisiología Reproductiva, Unidad de Investigación en Biología de la Reproducción, Facultad de Estudios Superiores Zaragoza, Universidad Nacional Autónoma de México, Ciudad de México, México; 3 Instituto de Ciencias, Benemérita Universidad Autónoma de Puebla, Puebla, México; 4 Vicerrectoría de Investigación y Estudios de Posgrado, Benemérita Universidad Autónoma de Puebla, Puebla, México; 5 Instituto de Fisiología, Benemérita Universidad Autónoma de Puebla, Puebla, México

**Keywords:** demyelination, seminiferous tubules, testosterone, LH

## Abstract

Presently, demyelinating diseases have been reported to affect the reproductive life of patients who suffer from them, but the progression of the alterations is unknown, especially in men. To better understand these effects, it is necessary to perform studies in animal models, such as the male *taiep* rat, which exhibits progressive demyelination of the central nervous system, altered kisspeptin expression at the hypothalamic level, and decreased luteinizing hormone, which could alter sperm quality and testicular diameter. Thus, the objective of the present study was to analyze the diameter of the seminiferous tubules, the sperm motility, and the testosterone levels of 90-day-old male *taiep* rats. The obtained results indicate that male *taiep* rats show an increase in testicular size accompanied by an increase in the diameter of the seminiferous tubules of the left testicle. There was also a decrease in progressive motility in sperm samples from the left epididymis of male *taiep* rats compared to the control group, with no changes in serum testosterone concentration. Therefore, we conclude that male *taiep* rats with central demyelination show altered testicular diameter and decreased motility in sperm from the left side. This type of studies serves as a basis for proposing possible reproductive strategies to improve the fertility and testicular function of men with demyelinating diseases of the central nervous system.

## Introduction

Multiple sclerosis is currently one of the most common demyelinating diseases (Franek et al., 2019; Rivera, 2017; Thompson et al., 2018), and its symptomatology and progession allows classifying it as relapsing-remitting, primary progressive, or secondary progressive (Thompson et al., 2018). In addition to motor problems related to myelin loss, reproductive problems have been reported in male patients with multiple sclerosis, such as erection and ejaculation problems (Cavalla et al., 2006; Wei and Lightman, 1997). These patients also show a decrease in testosterone concentration and hypogonadism (Glazer et al., 2018; Pakpoor et al., 2014). When evaluating the ejaculate of patients aged 18-55 years with relapsing-remitting multiple sclerosis, no significant differences were observed in volume, concentration, morphology, or progressive motility (D’Amico et al., 2020), although the authors do not rule out that this may be due to the size of the population analyzed.

In order to understand the changes derived from the demyelinating process at the reproductive level, it is necessary to have animal models that facilitate the study of these alterations. Among the animal models that resemble multiple sclerosis, there is the experimental autoimmune encephalomyelitis (EAE) model, where males have been reported to show alterations at the hypothalamic level, such as a down-regulation of GnRH mRNA and a decrease in testosterone resulting from a down-regulation of Star, Cyp11a1, Cyp17a1, and Hsd3b1/2 mRNA expression and StAR and Cpy11a1 protein expression in interstitial testicular cells (Milosevic et al., 2021, 2020). However, given that multiple sclerosis is an idiopathic disease with different courses, such as relapsing-remitting, secondary progressive, and primary progressive (Thompson et al., 2018), it is necessary to evaluate the alterations in other animal models that present demyelination of the central nervous system.

One model that can help us understand the changes that occur at the reproductive level during the demyelinating process is the *taiep* rat. This rat was obtained through selective crossbreeding of the Sprague-Dawley rat (SD) (Holmgren et al., 1989). The *taiep* rat is characterized by an initial hypomyelination that progresses to demyelination of the central nervous system, whose origin is a mutation in chromosome 9 of the tubb4A gene that causes the accumulation of microtubules in the smooth endoplasmic reticulum (Duncan et al., 2017, 1992; O’Connor et al., 2000; Song et al., 2001). The name *taiep* is an acronym of the progressive signs of tremor, ataxia, immobility, epilepsy, and paralysis of the hind limbs (Holmgren et al., 1989; Lunn et al., 1995). The *taiep* rat exhibits a down-regulation of IL-1b, IL-10, and TGFβ1, as well as astrocytosis at the level of the central nervous system (Leon Chavez et al., 2001; Soto-Rodriguez et al., 2015). In the case of male *taiep* rats, an increase in kisspeptin immunoreactive cell bodies has been observed at the hypothalamic level accompanied by a decrease in LH secretion compared to Sprague-Dawley rats (Muñoz de la Torre et al., 2022); these alterations could result in changes at the testicular and sperm level.

In the present study, we used 90-day-old male *taiep* rats to characterize LH and testosterone concentrations, testicular, epididymis, and seminal vesicle weight, alterations in the diameter of seminiferous tubules, and sperm motility. Banczerowski et al. showed that a right-sided lesion of the insular cortex results in a significant decrease in testosterone concentration in the left testicle (Banczerowski et al., 2001), and given that other studies have shown that the left testicle is more susceptible to cryptorchidism and variocele compared to the right one (Bogaert, 1997; Gerendai and Halász, 2001), it is possible to propose that there are differences in the function of these paired organs. Thus, in the present study we analyzed seminiferous tubule diameter and sperm motility considering each paired organ separately. This information will contribute to the knowledge of the alterations at the gonadal level that occur in an animal model with a demyelinating process.

## Materials and methods

### Animals

*Taiep* rats were obtained from the Animal Facility of the Institute of Physiology of the Benemérita Universidad Autónoma de Puebla. The *taiep* rat colony is maintained by crossing homozygous *taiep* rats with homozygous or F1 heterozygous *taiep* rats (Holmgren et al., 1989). All *taiep* rats used were obtained from crosses between homozygous *taiep* rats. Sprague-Dawley rats were used as a control because it is the strain of origin of the *taiep* rat and does not have reproductive alterations. Eighteen 90-day-old animals were used: 9 *taiep* and 9 SD. All rats were kept under controlled conditions of light-darkness (12-12 hours, lights turned on at 7 am) and temperature (22±2°C) in boxes with 3 individuals and free access to food and water.

At 90 days of age, the animals were weighed in a digital scale (Nahita, series 5041). All animals were sacrificed by pentobarbital sodium overdose (PiSA Agropecuaria, SA de CV) (63 mg/ml, 0.20 ml/100 g body weight) between 09:00 and 11:00 h. Blood was collected and kept at ambient temperature for 30 min and then centrifuged at 3500 rpm for 15 min. The serum was separated and stored at -20°C in microcentrifuge tubes until LH and testosterone quantification. The testicles, epididymides, and seminal vesicles were dissected, and every organ was weighed fresh in an analytical scale (Ohaus, AS120).

All experiments were performed according to the Mexican law of protection and treatment of animals and following the specifications in the official Mexican standard NOM-062-ZOO-1999. The experiment was approved by the committee for the care and use of laboratory animals of the Benemérita Universidad Autónoma de Puebla under protocol number 100274222-UALVIEP-20/1, reducing as much as possible the number of animals used and preventing their suffering.

### Testosterone and LH quantification

Free testosterone was quantified using ELISA kits following the manufacturer’s specifications (DRG International Inc., USA). The intra- and inter-assay variabilities for the testosterone kit were less than 4% and 9%, respectively, with a detection limit of 16ng/mL, LH levels were quantified using ELISA kits following the manufacturer’s specifications (Enzo Life Sciences, Farmingdale, NY, USA), which use a mouse anti-LH monoclonal antibody that recognizes human and rat LH. The intra-assay coefficient of variation was 5.3%, while the inter-assay coefficient was 15.5%. The sensibility of this assay is 0.612ng/mL with a confidence limit of 2. The absorbance values were measured with a microplate reader (ELx8’’, BioTek instruments, Inc, USA) at 450 nm and the final concentrations are reported in ng/mL.

### Diameter of seminiferous tubules

The testicles were fixed in 10% NBF, dehydrated, embedded in paraffin, and sectioned. A microtome was used to obtain 5mm-thick sections, which were placed on gelatinized slides. The sections were deparaffinized in xylene and rehydrated through 2 changes of 100% ethanol, followed by 95% ethanol, 70% ethanol, and water. All sections were stained with hematoxylin and eosin. Slides containing sections showing the initial, middle, and final parts of the organ were selected. We obtained 1080 images at 10X (540 images per strain, 60 images per rat, 30 images per testicle) using a Moticam camera (Motic®) and a Zeiss optical microscope (Germany). Where tubule diameter was determined (Laoung-On et al., 2021) using the software ImageJ 1.50i (ImageJ , 2023).

### Sperm count and motility

A cut was made in each epididymis tail and 10 mL of phosphate-buffered saline (PBS) were administered at 37°C for 1 minute to separate the spermatozoa. Subsequently, 100 μL of the sample were placed on a slide and covered. Five fields were selected and observed in a Zeiss microscope at 40x, and the spermatozoa present in each field were counted and classified into 3 motility categories (Table 1).

**Table 1:** Classification of sperm motility.

**Type of motility**	**Characteristics**
Progressive motile	Spermatozoa that show movement advancing in a certain direction.
Non-progressive motile	Spermatozoa that show movement but do not advance.
Non-motile	Spermatozoa that do not move.

### Statistical analysis

The obtained data were analyzed with a Shapiro-Wilk normality test. Diameter comparisons were made using a two-way ANOVA. (α = 0.05). The factors used in the ANOVA were “Strain”, *taiep* and SD rats, and “Side”, left and right. All pairwise multiple comparison procedures were performed using the Holm-Sidak method for group comparisons. The comparisons of LH and testosterone serum levels and organ weight were made with a Student’s t-test. Motility percentages were analyzed with a Z-test. Differences in probabilities equal or lower than 0.05 were considered statistically significant. All analyses were performed in SigmaPlot V.11.0 (Systat Software, 2023).

## Results

### Body and organ weight

Male *taiep* rats of 90 days of age showed decreased body weight compared to male SD rats of the same age (Table 2). Due to this difference in body weight between strains, this study used the relative weight to analyze organ weight, defined as milligrams per 100 gr of body weight (mg100g/BW). The relative weight of the testicles, epididymides, and seminal vesicle of *taiep* rats was significantly higher than the relative weight of the same organs of SD rats (Table 2).

**Table 2:** Body and organ weight of male taiep and SD rats of 90 days of age.

**Variables**	**taiep**	**SD**	**df**	**t**	**p-value**
**Body weight (g)**	297.9±3.6	427.8±9.5	129.9	12.837	<0.001*
**Seminal vesicle (mg/100gBW)**	435.5±30.5	301.7±26.1	133.799	-3.335	0.005*
**Testicles (mg/100gBW)**	1048.6±26.5	843.7±26.2	-204.864	-5.489	<0.001*
**Left**	516.04±14.9	409.02±9.3	-107.021	-6.095	<0.001*
**Right**	521.9±11.8	423.7±14.6	-98.241	-5.227	<0.001*
**Epididymides (mg/100gBW)**	376±8.63	298.6±17.3	-77.415	-4.011	0.001*
**Left**	188.6±6.6	142.5±6.1	-46.162	-5.131	<0.001*
**Right**	185.6±4.5	151.8±10.3	-33.742	-3.001	0.010*

Data are expressed as the mean±SEM with units in milligrams per 100g of body weight (mg/100gBW). Significant values are marked with *; Student’s t-test, taiep vs SD. Abbreviations: SD: Sprague-Dawley.

### LH and testosterone concentrations

**Figure 1:**
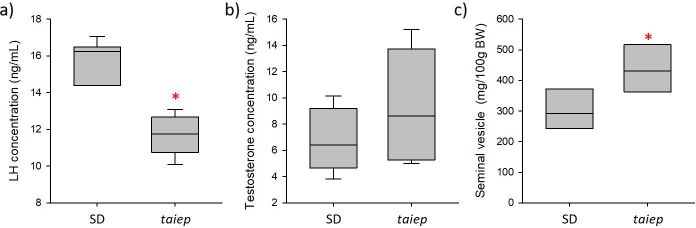
Graphs showing a) luteinizing hormone (LH) concentration, b) testosterone concentration, and c) seminal vesicle relative weight in *taiep* and SD rats. Abbreviations: mg/100g BW: milligrams per 100 g body weight. * indicates significant differences between strains (*taiep* vs SD), p<0.05 T-student test.

Male *taiep* rats showed a significant decrease in serum LH concentrations compared to SD rats (11.65±1.02 ng/mL vs 15.85± 0.98 ng/mL; df: -4.19, t:- 2.967, p=0.009, Figure 1). No significant changes in serum testosterone concentrations were observed in *taiep* rats compared to SD rats (9.23±1.38 ng/mL vs 6.917±0.774; df: 2.32, t:1467, p=0.162, Figure 1).

### Analysis of diameter of seminiferous tubules

**Figure 2:**
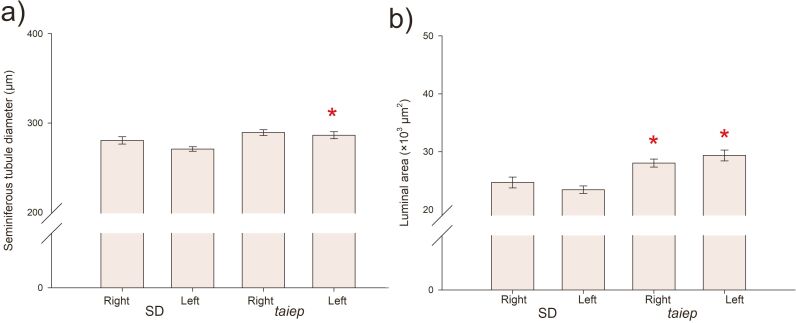
Seminiferous tubule diameter. The graph shows the mean SEM of the diameter. * Indicates significant differences between strains (*taiep* vs SD), p<0.05 Holm-Sidak method. Abbreviations: SD: Sprague-Dawley.

The two-way ANOVA of the diameter of the seminiferous tubules showed significant differences between strains (F= 11.495; p<0.001), with no significant differences between sides (F= 3.134; p=0.077), and the interaction between the two factors was also not significant (Strain x Side F= 0.900; p=0.343). The diameter of the seminiferous tubules of *taiep* rats was larger compared to that of SD rats (SD 275.69±2.62 vs *taiep* 287.77±2.41, df =12.072, p<0.001). The seminiferous tubules from the left testicle of *taiep* rats showed a larger diameter than those from the left testicle of SD rats (Figure 2).

### Sperm motility

No significant changes were observed in total sperm count or sperm count by side (Table3, a). There were no observed differences in the total percentage of progressive motile, non-progressive motile, and non-motile spermatozoa between *taiep* and SD rats (Table 3, b). When analyzing sperm motility by side, the left epididymis of *taiep* rats was observed to have a lower percentage of progressive motile spermatozoa and an increase in non-motile spermatozoa compared to the right side. When comparing the percentage of progressive motile spermatozoa obtained from the left epididymis of *taiep* rats, there is a decrease compared to SD rats, which is compensated by the right epididymis, since it shows an increase in this sperm category (Table3, c). No difference was observed in the percentage of non-progressive motile spermatozoa. Finally, when analyzing the percentage of non-motile spermatozoa, *taiep* rats showed an increase on the left side and a decrease on the right side compared to SD rats (Table 3, c).

**Table 3:** Number of spermatozoa and percentage of spermatozoa in each sperm motility category obtained from the epididymides of taiep and SD rats.

**a) Number of spermatozoa**												
	***taiep***		***SD***			**df**		**t**			**p-value**	
**Total**	288.4±25.64		283.9±32.27			-4.5		-0.109			0.914	
**Left side**	273.9±18.35		278±18.95			4.111		0.156			0.878	
**Right side**	303±49.04		289.9±63.69			-13.111		-0.163			0.872	
**b) % of spermatozoa in each motility category**												
**%**	***taiep***		***SD***					***z***			***p-value***	
**Progressive motile**	28.43		31.81					0.79			0.430	
**Non-progressive motile**	25.47		29.43					0.968			0.333	
**Non-motile**	46.10		38.76					1.69			0.091	
**Total**	100%		100%									
**c) % of spermatozoa in each motility category by side**												
**%**	***taiep***			***SD***			***Z test p-value***					
							***taiep vs SD***			***Left Vs Right***		
	***Left***	***Right***		***Left***	***Right***		***Left***		***Right***	***taiep***		***SD***
**Progressive motile**	12.69	43.49		35.25	28.32		<0.001*		<0.001*	<0.001*		0.098
**Non-progressive motile**	25.14	25.78		29.34	29.53		0.304		0.360	0.934		0.966
**Non-motile**	62.17	30.73		35.41	42.15		<0.001*		0.006*	<0.001*		0.125
**Total**	100	100		100	100							

The data in a) are the mean ± SEM, showing the total value and the value per epididymis. Sections b) and c) show the percentage of spermatozoa in each motility category. Significant values are marked with *. Abbreviations: SD: Sprague-Dawley.

## Discussion

In the present study, we do not discard the possibility that the loss of body weight and the alterations in organ weight in *taiep* rats could be the result of the crossbreeding to obtain this animal model, which would have to be evaluated as an independent factor in subsequent analyses. At the moment, we can suggest that the decrease in body weight in our animal model is related to inflammatory alterations such as the down-regulation of TGFβ1 at the central level (Soto-Rodriguez et al., 2015). A decrease in TGFβ1 at the hypothalamic level has been reported to cause an increase in locomotor activity accompanied by increased O2 consumption and CO2 production, along with a reduction in interscapular brown adipose tissue, resulting in weight loss (Mendes et al., 2018). Weight loss has also been observed in other animal models of demyelination (Milosevic et al., 2020) and patients with multiple sclerosis (Bergamaschi et al., 2006; Reder et al., 1994) and Huntington’s (Aziz et al., 2008; Djousse et al., 2002).

Male *taiep* rats showed a decrease in plasma LH concentrations, which is similar to that observed in the murine model of EAE (Milosevic et al., 2020) and patients with multiple sclerosis (Safarinejad, 2008). This reduction in LH could be a consequence of the proinflammatory environment exhibited by the *taiep* rat at the level of the central nervous system, characterized by reduced remyelination-stimulating factors, such as FGF2 and CXCL1, and the down-regulation of IL-1β, IL-10, TGFβ1 at the level of the cerebellum and brainstem (Soto-Rodriguez et al., 2015; Soto-Rodríguez et al., 2012), possibly resulting in the inhibition of GnRH secretion. Wu and Wolfe reported that GnRH secretion is inhibited in the presence of interleukins such as IL10 (Wu and Wolfe, 2012). If this were the case, it is possible to hypothesize that the presence of proinflammatory markers, such as interleukins, in the brain of the *taiep* rat would modify the pattern of GnRH secretion, resulting in an alteration in LH secretion (Han et al., 2020). Future studies could focus on showing the presence of changes in GnRH secretion. For this, we propose the administration of buserelin, a GnRH analogue that has been observed to result in increased LH secretion in EAE models (Milosevic et al., 2020), and analyze whether administering it in *taiep* rats improves the secretion of this pituitary hormone and the reproductive parameters.

A decrease in testosterone associated with a decrease in LH (Oduwole et al., 2021) is not observed in the *taiep* rat. Given that Leydig cells synthesize testosterone in the presence of LH stimulation (Zirkin and Papadopoulos, 2018), it is possible to suggest that testosterone levels are maintained by an increase in the number of Leydig cells or by an increase in the availability of LH receptors in these cells, which should be evaluated in the future. An increase in the presence of LH receptors could be a consequence of increased follicle-stimulating hormone (FSH) secretion, since the administration of this hormone in rats has been reported to increase gonad size and LH receptor number in Leydig cells and is able to maintain spermatogenesis in the absence of LH (O’Shaughnessy et al., 2010; Oduwole et al., 2021; Vihko et al., 1991).

In the case of the increase in reproductive organ weight, it could be caused by the interaction of several factors such as testosterone, FSH, and glucocorticoids. Justulin et al. reported an increase in seminal vesicle size in castrated rats treated with testosterone (Justulin et al., 2006). Thus, in the case of the *taiep* rat, an increase in seminal vesicle size could be a consequence of the testosterone levels, which tend to increase, and it could be sufficient to cause this effect. Regarding the increase in gonad size, an increase in insulin-like growth factor (IGF1) signaling, which triggers PI3K/AKT- and MAPK-mediated intracellular signaling pathways involved in FSH proliferative action, has been observed during development (Cannarella et al., 2018). In rodents, the administration of FSH during puberty causes an increase in testicular volume (Nurmio et al., 2012; O’Shaughnessy et al., 2010; Vihko et al., 1991), and thus it is possible to suggest that, in our animal model, there could be an increase in IGF1 activity that together with FSH would produce this increase in gonad size.

The increase in testicular size in the *taiep* rat was accompanied by an increase in the diameter of the seminiferous tubules. This increase in diameter could be a consequence of an increase in FSH concentration (O’Shaughnessy et al., 2010; Oduwole et al., 2021). Banczerowski et al. showed that a right-sided lesion of the insular cortex results in a significant decrease in testosterone concentration in the left testicle (Banczerowski et al., 2001), and given that other studies have shown that the left testicle is more susceptible to cryptorchidism and varicocele compared to the right one (Bogaert, 1997; Gerendai and Halász, 2001), it is possible to propose that there are differences in the function of these paired organs. When performing the analysis by side, the seminiferous tubules of the left testicle clearly show the highest degree of alteration, which agrees with the idea that the left testicle is more sensitive to changes such as hemicastration (Brown and Chakraborty, 1991; Frankel et al., 1989) and lesion of the insular cortex (Banczerowski et al., 2001).

Given that patients with MS show an alteration in the neural connectivity associated with motor control due to myelin loss and increased inflammation (Peterson and Fling, 2017), it is possible to suggest that in *taiep* rats that exhibit a demyelinating process with the presence of inflammatory cytokines (Soto-Rodriguez et al., 2015) accompanied by reactive astrocytosis (Leon Chavez et al., 2001) synaptic transmission is affected at the level of the brainstem, which would modify the transmission of information through the hypothalamic-testicular neural pathway (Lee et al., 2002) thus, impacting testicular function and showing the changes that we observed in the present study.

It is currently recognized that there is communication between the central nervous system and the gonads. In the case of males, the gonads are connected to the central nervous system by the superior (SSN) and inferior (ISN) spermatic nerves (Lee et al., 2002). It has been shown that surgical denervation of the SSN and ISN in pubertal rats results in the loss of spermatogenesis when the rats reach the adult stage, where there is a decrease in seminiferous tubule area and an increase in the apoptosis of round spermatogonia and Leydig cells (Huo et al., 2010). Furthermore, sectioning of the SSN and ISN in adult rats results in a partial loss of spermatogenesis and a progressive loss of proliferating spermatogonia and preleptotene spermatocytes in the tissue of the seminiferous tubules (Chow et al., 2000). This experimental evidence shows the importance of nerve communication and testicular physiology.

Although there was an increase in the diameter of the seminiferous tubules, there were no changes in sperm count in the epididymis of *taiep* rats compared to SD rats, as well as no changes in sperm motility when evaluating the samples obtained from both epididymides, which would be equivalent to an ejaculate, and this is similar to what is observed in patients with multiple sclerosis (D’Amico et al., 2020). However, when performing the analysis considering the side of the epididymis, there was a higher presence of non-motile spermatozoa in the left epididymis and an increased percentage of progressive motile spermatozoa in the right epididymis. This effect could be caused by an increase in the time that sperm spend in the epididymis (Fernandez et al., 2008). The information that the left epididymis receives from the nervous system is possibly deficient due to the demyelination that occurs in the *taiep* rat at the level of the basal ganglia and the cerebellum (Garduno-Robles et al., 2020; Lopez‐Juarez et al., 2021), which would cause a reduction in the peristaltic movements (Sakakibara, 2021) that allow the passage of sperm.

In the present study, we show that *taiep* rats have a decrease in LH concentration, which has also been observed both in men with MS (Safarinejad, 2008) and the EAE model (Milosevic et al., 2021). Moreover, as in men (Safarinejad, 2008), our model shows alteration in sperm motility. In contrast with the EAE model (Milosevic et al., 2021) and men (Safarinejad, 2008), we did not observe changes in testosterone concentration. However, our model showed an increase in testicular and accessory reproductive organ weight, which shows that the *taiep* rat is an interesting model to continue the analysis of reproductive alterations in males with demyelination of the central nervous system.

## Conclusion

The results of the present study show that adult male *taiep* rats, which have demyelination of the central nervous system, show an alteration in the diameter of the seminiferous tubules. These results allow concluding that the demyelinating process has a stronger effect on the left testicle and epididymis. Our results contribute to the knowledge about testicular alterations in a model of demyelination of the central nervous system. We propose the *taiep* rat as a model to continue analyzing reproductive alterations in males over time, which will help to understand the reproductive alterations in men who suffer from this type of disease.
